# The interplay between prenatal liver growth and lung development in congenital diaphragmatic hernia

**DOI:** 10.3389/fped.2022.983492

**Published:** 2022-09-26

**Authors:** Katherine C. Ott, Michael Bi, Federico Scorletti, Saad A. Ranginwala, William S. Marriott, Jose L. Peiro, Beth M. Kline-Fath, Amir M. Alhajjat, Aimen F. Shaaban

**Affiliations:** ^1^Department of Surgery, Ann and Robert H. Lurie Children's Hospital, Chicago Institute for Fetal Health, Northwestern University Feinberg School of Medicine, Chicago, IL, United States; ^2^Cincinnati Fetal Care Center, Cincinnati Children's Hospital, University of Cincinnati College of Medicine, Cincinnati, OH, United States; ^3^Neonatal Surgical Unit, Medical and Surgical Department of the Fetus, Newborn and Infant, Bambino Gesù Children's Hospital, Rome, Italy

**Keywords:** congenital diaphragm hernia, liver development, in utero imaging, liver growth, pulmonary hypoplasia, pulmonary hypertension

## Abstract

**Objective:**

Liver herniation is a known risk factor for increased severity in CDH and is associated with clinically significant pulmonary hypoplasia and pulmonary hypertension. Better studies are needed to understand the growth of the herniated liver compared to the liver that remains in the abdomen and how this liver growth then affects lung development. Serial hi-resolution fetal MRI enables characterization of liver growth throughout gestation and examination of macroscopic features that may regulate liver growth. Here, we hypothesized that the nature of liver herniation affects liver growth and, in turn, affects lung growth.

**Methods:**

Clinical data were retrospectively collected from consecutive cases of prenatally diagnosed isolated left-sided or right-sided CDH from June 2006 to August 2021. Only those cases with MRI lung volumetry for both mid-gestation and late-gestation time points were recruited for analysis. Cases with fetal chromosomal abnormalities and other major structural abnormalities were excluded. Fractional liver volume and liver growth was indexed to estimated fetal weight and compared to lung growth.

**Results:**

Data was collected from 28 fetuses with a left liver-down CDH (LLD), 37 left liver-up CDH (LLU) and 9 right liver-up CDH (RLU). Overall, RLU fetuses had greater overall and fractional (intra-thoracic vs. intra-abdominal) liver growth when compared to LLD and LLU fetuses. Additionally, intra-thoracic liver growth was consistently slower than intra-abdominal liver growth for either right- or left-sided CDH. When the liver was not herniated, a positive correlation was seen between liver growth and lung growth. However, when the liver was herniated above the diaphragm, this positive correlation was lost.

**Conclusion:**

Right-sided CDH fetuses exhibit greater liver growth compared to left-sided CDH. Liver herniation disrupts the normal positive correlation between liver and lung growth that is seen when the liver is entirely within the abdomen.

## Introduction

Congenital diaphragmatic hernia (CDH) occurs in 1 in 2,500 to 1 in 5,000 live births and is associated with significant neonatal morbidity and mortality (1–4). Advances in prenatal imaging permit risk-stratification for affected fetuses using multiple static indices including o/e LHR, o/e TLV, PPLV, %LH and LiTR (5–14). An assessment of these parameters facilitates prenatal counseling, delivery planning and, more recently, determines eligibility for prenatal intervention (15, 16). There is an inherent lack of precision in these indices as they are static, rather than trended throughout gestation. Dynamic variables are needed to better understand outcomes to the fetus, particularly late in gestation when liver growth above the diaphragm may adversely affect lung growth. Understanding factors that affect lung development throughout gestation in CDH will aid in the development of more precise dynamic predictors of patient outcomes.

Liver herniation carries a worse prognosis in CDH and is associated with pulmonary hypoplasia and pulmonary hypertension (17–20). In the experimental nitrofen-induced CDH rat models, larger liver volumes were seen in mice with CDH compared to controls (21). Additionally, larger intrathoracic liver volumes were seen in mice with larger diaphragmatic defects which then correlated with more severe pulmonary hypoplasia (21). In humans, liver herniation into the chest is an indicator of worse prognosis (8–10). The fraction of liver herniation has been shown to predict need for ECMO support and overall survival (6–13, 17, 22). Additionally, the ratio of the intrathoracic liver volume to total thoracic volume (LiTR) predicted postnatal survival independently from o/e TLV (11). When looking at multiple modes of liver and lung assessment, a combination of o/e TLV and %LH provided the best predictive measure for ECMO use and mortality (9). Given the rigid structure of the chest, growth of the intrathoracic liver throughout gestation likely has a negative impact on fetal lung development. Yet, no study in CDH fetuses exists to directly challenge this postulate. Serial hi-resolution fetal MRI enables simultaneous characterization of liver and lung growth throughout gestation and illuminates the consequences of these seemingly reciprocal developmental processes in the CDH fetus. In this study, serial MRI is used to define the relationship of liver growth to lung growth in both left and right sided CDH in *utero*.

## Materials and methods

### Study population

Clinical data was collected from cases of both prenatally diagnosed left-sided and right-sided CDH at the Cincinnati Fetal Center and the Chicago Institute for Fetal Health from June 2006 to August 2021 with institutional review board approval (IRB # 2011-2626 and IRB #2020-3050). Only those with complete information on MRI-derived lung volume for both mid-gestation (18–30 completed weeks gestation) and late-gestation (after 30 completed weeks gestation) time points were included in the analysis. Exclusion criteria were fetal chromosomal abnormalities and other major structural abnormalities. No patients in the study population underwent prenatal intervention. Patients were divided into either left-sided CDH with liver entirely down in the abdomen (LLD), left- or right-sided CDH with liver up (LLU or RLU). All patients with a right-sided CDH had some degree of liver herniation into the chest. Gestational age was determined from a well-defined last menstrual period and/or by sonographic biometry in the first trimester of pregnancy.

### Prenatal MRI evaluation

Fetal Ultrafast MRI study was performed using a 1.5-T scanner (Horizon; General Electric Medical System, Milwaukee, WI and Ingenia; Phillips Healthcare, Holland) without maternal or fetal sedation. A torso-phased array coil was used to optimize all studies. The MRI protocol included two sequences: a single-shot fast-spin echo T2-weighted sequence with matrix of 224 × 192, a field of view of 30 or less, and a gradient-echo T1-weighted sequence with a matrix of 256 × 192, a field of view of 30–35. The T2 weighted imaging was performed at a slice thickness of 3 or 4 mm and T1 sequences at 5 mm. The corresponding areas of liver down and liver up were determined on each MRI section by using a free-form region of interest on a Vitrea workstation (Vital Images Inc, Minnetonka, MN). Delineation of the liver up and liver down on the ipsilateral site of defect was determined by mirroring the contour of the contralateral diaphragm. Liver volumes were then calculated from T1-weighted images acquired in the coronal plane, which were sequential and without motion, and the summation of the liver areas from all coronal images were multiplied by the thickness of the section. All MRI scans were reviewed and reported by 2 independent fetal radiologists with combined 40 years of experience. Using this methodology, intra- and inter-reviewer reproducibility has been demonstrated to be moderate-to-good for liver volumes above the diaphragm and good-to-excellent for liver volumes below the diaphragm after standardization of the technique (23).

**Figure 1:**
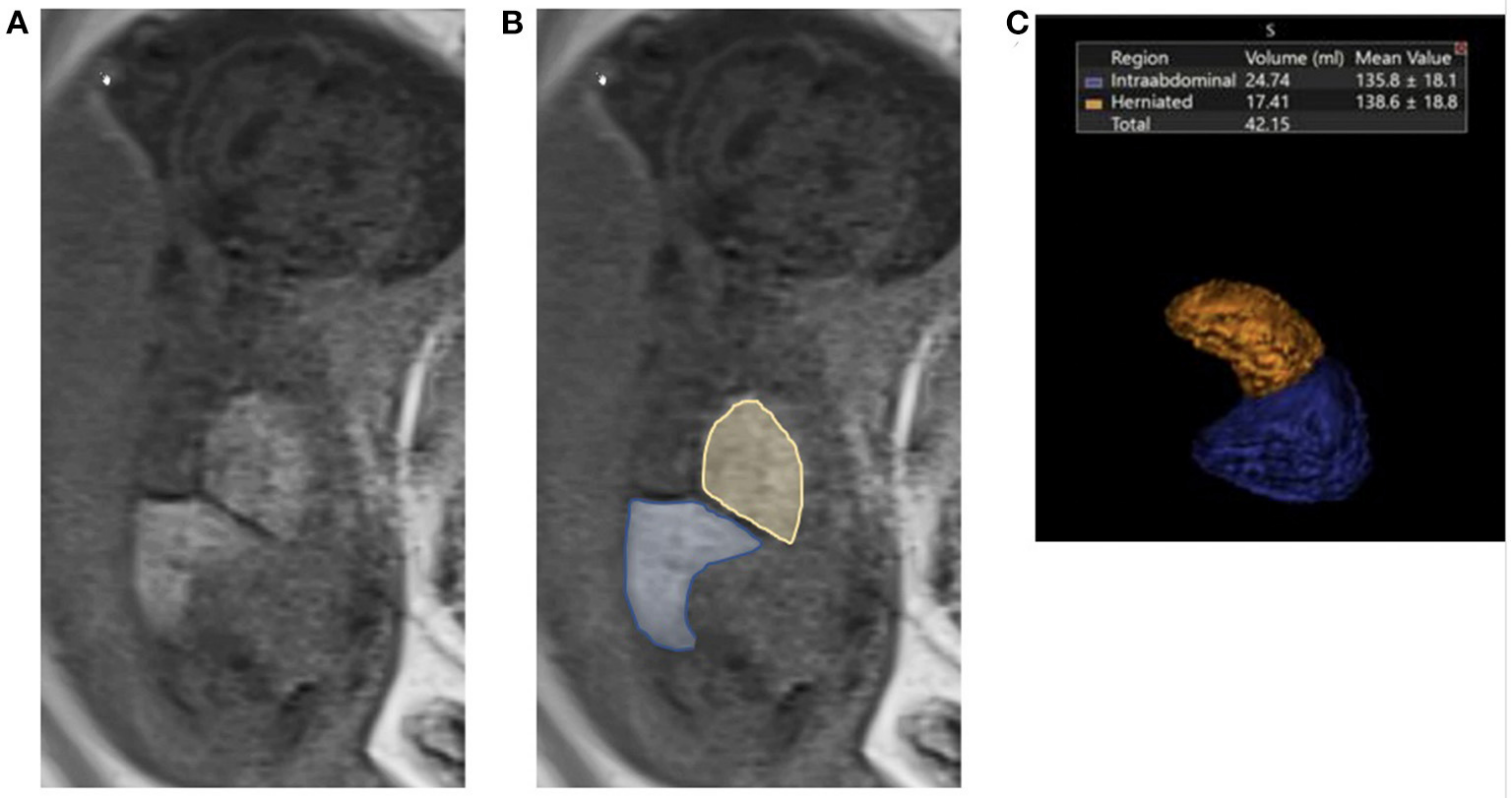
Fetal liver volumetry using T1 MRI images. **(A)** Liver down (blue) and liver up (yellow) volumes were determined on each MRI section by using a free-form region of interest on a Vitrea workstation (Vital Images Inc, Minnetonka, MN). **(B)** Delineation of the liver up and liver down on the ipsilateral site of defect was determined by mirroring the contour of the contralateral diaphragm. Liver volumes were then calculated in the T1-weighted coronal plane by using sequences that allowed complete imaging of the liver and without artifacts from motion and the summation. **(C)** Three dimensional representation of the liver volumes from the same patient.

The following MRI findings were collected for analysis: LU (liver up), LD (liver down), and LT (liver total) in milliliters (ml). Examples of T1 MRI images from a left sided CDH fetus with liver herniation are shown in Figure 1 both before (A.) and after (B.) tracing of the liver. Figure 1C. shows a three dimensional representation of the liver volumes from the same patient. The presence of intrathoracic liver was defined as any portion of liver detected in the thoracic cavity, identified by MRI study. LT was calculated by the summation of the LD and LU (measured in mL). To track liver growth relative to fetal size, fractional and total liver volumes and liver growth were also normalized to estimated fetal weight (EFW) measured late in gestation (EFW2) to normalize the liver growth against the growth of the fetus overall. MRI lung volumes were calculated from coronal T2 weighted non-motion sequences and also obtained by the 2-D areas multiplied by the thickness of the slice. Total lung volume was then obtained *via* the summation of the right and left lung volumes (in ml). The o/e TLV volumes were calculated according to the method outlined by Rypens et al. (24). Lung growth was measured as ΔTLV/EFW2/Δ*T*.

### Statistical analysis

Graphs were created in Graphpad Software Version 9.4.0 (San Diego, CA) to characterize liver volumes and liver growth. Linear regression was calculated assessing liver growth and lung growth. Statistical analyses were performed with IBM Corp. © SPSS Statistics, Version 28.0.1.0 (Armonk, NY).

## Results

### Cohort characteristics

Baseline maternal and fetal characteristics, growth parameters and o/e LHR are presented in Table 1. Maternal age at delivery, birthweight, and gestational age at delivery were similar between groups. Mid gestational age at time of first MRI and late gestational age at time of second MRI were also similar between groups. Estimated fetal weight was measured for all groups at both mid and late gestation. There was no significant difference in baseline growth parameters between the groups. In order to measure disease severity, o/e LHR was measured. o/e LHR was higher in the liver down group compared to the liver up groups. There was no significant difference between the liver up groups.

**Table 1:** Prenatal CDH cohort characteristics.

	**LLD**	**LLU**	**RLU**	***p*- value**
*N*	28 (38%)	37 (50%)	9 (12%)	
Maternal age (years)	28.8 ± 4.8	27.8 ± 6.0	28.9 ± 7.7	0.8
Birth weight (g)	3059 ± 439	2707 ± 446	3034 ± 284	0.1
Mid-gestation age (weeks)	24.6 ± 2.7	25.0 ± 2.7	24.9 ± 2.6	0.6
Late-gestation age (weeks)	34.1 ± 0.6	34.0 ± 0.7	33.1 ± 1.1	0.2
Gestational age at delivery (weeks)	38.3 ± 0.9	37.6 ± 1.3	37.8 ± 0.9	0.6
o/e LHR (%)	37.6 ± 13.1	23.8 ± 6.8	19.5 ± 10.6	<0.01

Table compares clinical characteristics, of study population groups.

### Liver volumes in right-sided CDH lower in mid-gestation and higher in late-gestation

A comparison of static liver volumes normalized to estimated fetal weight between the groups (LLD, LLU, and RLU) is summarized in Table 2. When normalized to EFW, RLU fetuses had lower mid-gestation and higher late-gestation liver down volumes when compared to LLD and LLU fetuses, as shown in Table 2. LLU fetuses also had lower liver-down volumes compared to LLD fetuses at both time points. RLU fetuses had higher late-gestation liver up volumes when normalized to EFW compared to LLU fetuses. Taken together, these findings show that RLU fetuses had a higher intrathoracic liver volume and a higher total liver volume when compared to both LLU and LLD fetuses despite having a lower total liver volume in mid-gestation.

**Table 2:** Liver volumes in right-sided CDH lower in mid-gestation and higher in late-gestation.

	**LLD**	**LLU**	**RLU**	***p*-value all**	***p*-value LLD-LLU**	***p*-value LLD-RLU**	***p*-value LLU-RLU**
Mid-LU/EFW1 (ml/kg)	–	13.4 ± 5.6	10.0 ± 4.2	–	–	–	0.07
Mid-LD/EFW1 (ml/kg)	68.8 ± 18.4	45.8 ± 13.1	29.0 ± 12.3	**<0.001**	**<0.001**	**<0.001**	**0.005**
Mid-LT/EFW1 (ml/kg)	68.8 ± 18.4	59.2 ± 13.7	39.0 ± 16.6	**<0.001**	**0.03**	**<0.001**	**0.010**
Late-LU/EFW2 (ml/kg)	–	12.6 ± 5.4	33.5 ± 8.0	–	–	–	**<0.001**
Late-LD/EFW2 (ml/kg)	56.0 ± 12.1	40.2 ± 8.4	60.6 ± 14.4	**<0.001**	**<0.001**	.**04**	**0.004**
Late-LT/EFW2 (ml/kg)	56.0 ± 12.1	52.8 ± 10.3	94.2 ± 24.4	**<0.001**	0.3	**0.001**	**0.001**

Comparison of liver volume (liver up/down/total) and liver volume normalized against estimated fetal weight (EFW) among LLU, LLD, RLU CDH patients. All variables assessed at mid-gestation and late-gestation. Bold values indicate *p* < 0.05.

### Faster liver growth in right sided CDH compared to left

**Figure 2:**
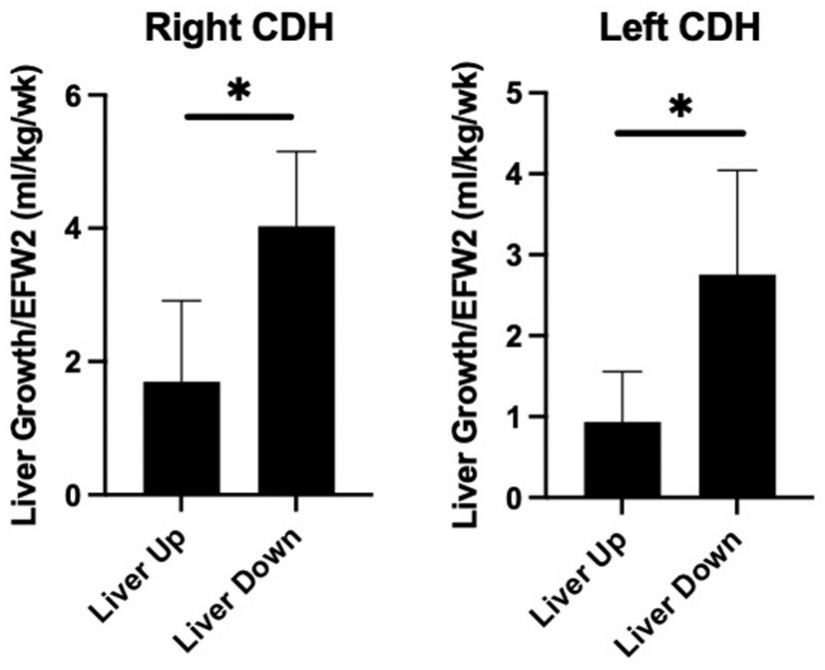
Greater liver growth below the diaphragm in prenatal CDH. Comparison of fractional liver growth (liver up and liver down) normalized to late gestation estimated fetal weight in right and left sided CDH fetuses. As shown, liver growth below the diaphragm is greater than the growth above the diaphragm in both right and left sided CDH (*p < 0.05).

Table 3 compares liver growth among the groups. When normalized to EFW, RLU fetuses had faster liver growth above the diaphragm than LLU fetuses. RLU fetuses had significantly more intrathoracic liver growth compared to LLU fetuses. RLU fetuses also had more liver down growth compared to LLU fetuses but similar liver down growth when compared to LLD. Total liver growth was also greater in the RLU fetuses when compared to both the LLU and LLD fetuses. Figure 2 shows that liver growth below the diaphragm was greater than liver growth above the diaphragm when normalized to EFW for both right- and left-sided CDH fetuses.

**Table 3:** Greater liver growth in right-sided CDH compared to left.

	**LLD**	**LLU**	**RLU**	***p*-value all**	***p*-value LLD-LLU**	***p*-value LLD-RLU**	***p*-value LLU-RLU**
Growth-LU/EFW2 (ml/kg/wk)	–	0.9 ± 0.6	1.7 ± 1.2	–	–	–	**0.01**
Growth-LD/EFW2 (ml/kg/wk)	3.9 ± 1.3	2.8 ± 1.3	4.0 ± 1.1	0.4	**0.001**	0.7	**0.01**
Growth-Total/EFW2 (ml/kg/wk)	3.9 ± 1.3	3.7 ± 1.5	5.7 ± 2.2	**0.003**	0.6	**0.005**	**0.002**

Table compares the growth of liver volume (liver up/down/total) and growth of fetal liver volume normalized against estimated fetal weight in LLU, LLD, RLU CDH patients. Significant differences are seen in the growth of liver up and liver down between LLU and RLU when normalized to EFW (*p* < 0.05). RLU fetuses display significantly more total liver growth compared to both LLU and LLD (*p* < 0.05). Bold values indicate *p* < 0.05.

### Intrathoracic liver growth adversely affects lung growth

**Figure 3:**
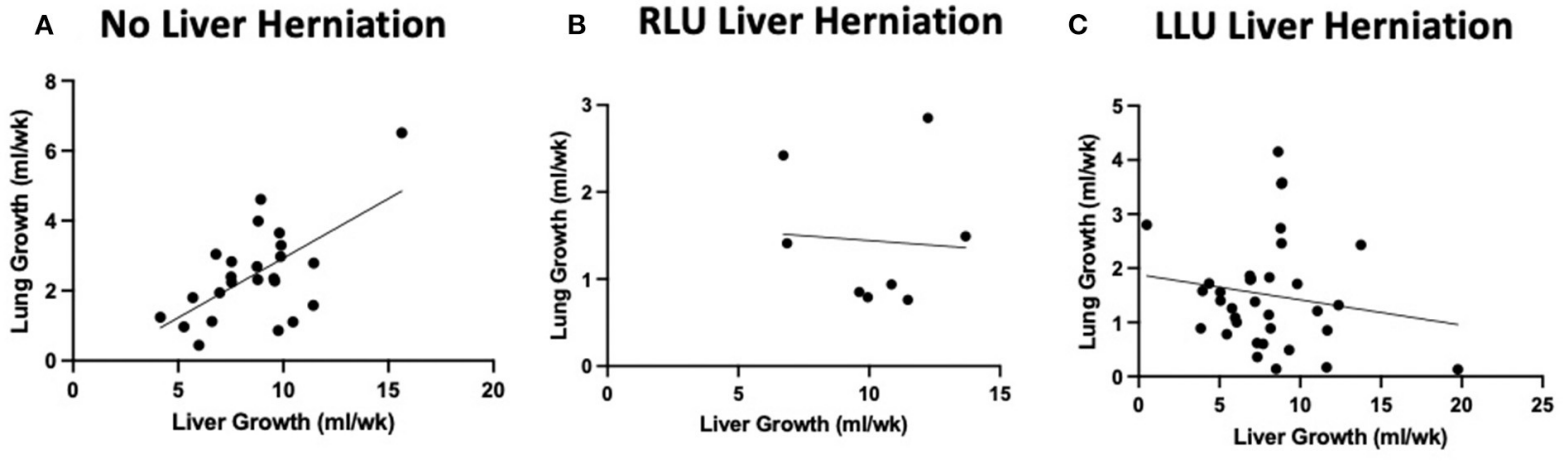
Liver herniation alters normal positive correlation between liver growth and lung growth. Graphical relationship between total liver growth and total lung growth in cases without liver herniation (LLD) and with liver herniation (RLU and LLU). **(A)** Positive correlation between total lung growth and total liver growth when the liver is not herniated above the diaphragm (*R*^2^ = 0.38). **(B,C)** Loss of positive correlation between lung growth and liver growth when liver is herniated above the diaphragm in both RLU and LLU groups.

**Figure 4:**
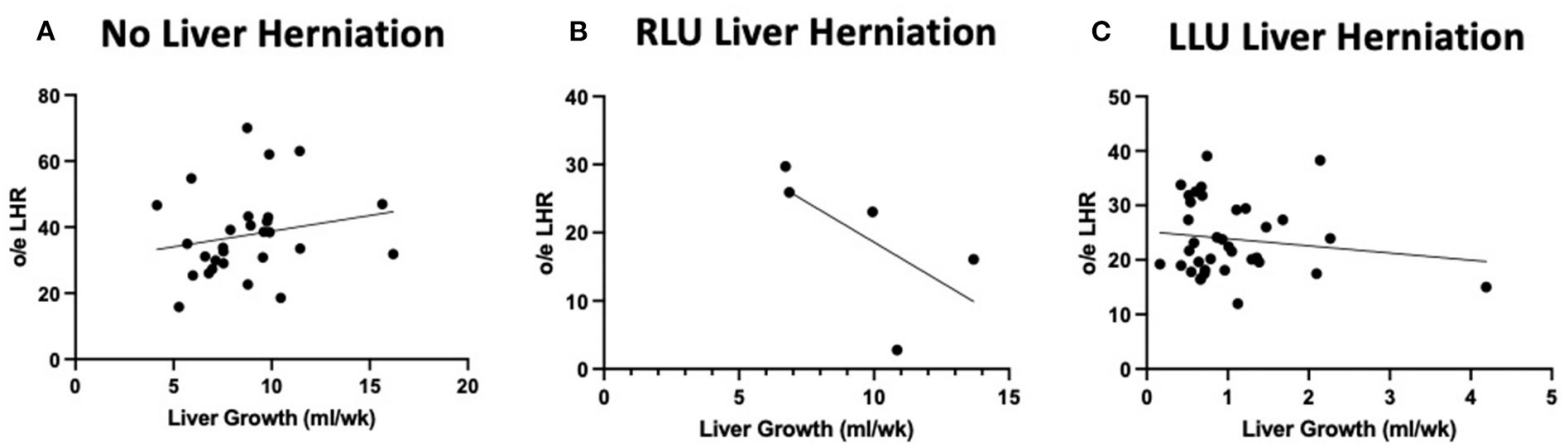
Liver growth correlations to o/e LHR in both herniated and non herniated groups. Graphical relationship between total liver growth and o/e LHR in cases without liver herniation (LLD) and with liver herniation (RLU and LLU). **(A)** No correlation between total o/e LHR and total liver growth when the liver is not herniated above the diaphragm. **(B)** A negative correlation between o/e LHR and liver growth when liver is herniated above the diaphragm in RLU group (R^2^ = 0.42). **(C)** No correlation between o/e LHR and liver growth when liver is herniated above the diaphragm in LLU group.

Figure 3 shows the relationship between liver growth and lung growth in fetuses with and without liver herniation. Lung growth correlated positively with liver growth for fetuses without liver herniation (*R*^2^ = 0.38; *p* < 0.05). However, the positive correlation between lung growth and liver growth was lost when the liver was herniated above the diaphragm. Liver growth was not associated with o/e LHR in the liver down group. Liver growth was correlated with lower o/e LHR in the RLU group (*R*^2^ = 0.42) however in the small sample size this association is not significant (Figure 4). o/e LHR also down trended with increasing liver growth in the LLU group but this correlation was also not significant.

## Discussion

In the current study, utilizing serial MRI, the relationship between liver and lung growth in *utero* is described. The presence of a right-sided diaphragmatic defect was associated with faster intrathoracic and overall fetal liver growth albeit in a small sample size. Liver growth below the diaphragm was greater than liver growth above the diaphragm independent of the side of the defect. Liver growth in CDH fetuses without liver herniation was correlated with increasing lung growth but this association was lost in cases with liver herniation above the diaphragm.

An understanding of how the liver grows in *utero* is limited despite many efforts both in animal models and humans. Under normal conditions, the total liver mass is limited by total body mass suggesting that liver size is determined physiologically by the size of the abdominal cavity (25). This relationship to body mass is also supported with the observation that liver ceases to regenerate after resection once the normal liver volume is achieved (26). Changes in the extracellular matrix changes and growth factor release also play a role in determining timing and degree of liver growth (27–29). The mechanisms regulating liver development in normal gestation are not well understood. Similarly, the regulation of liver growth under abnormal conditions such as in CDH has not been studied.

The current findings reveal that the side of the CDH affects the total and intrathoracic liver growth suggesting that the physical relationship to the diaphragm is a key determinant of liver growth. Additionally, liver growth is greater below when compared to above the diaphragm. This finding parallels findings from a recent study in a fetal rabbit model of CDH (30). The authors found that the intra-thoracic liver weight was higher in right-sided CDH (30). They also showed that there was corresponding lower lung weight in both left- and right-sided CDH when the liver was growing in the chest (30). The authors postulated that these differences could be due to lower compartmental pressures when the defect is directly over the liver allowing for increasing liver growth. Additionally, studies in the nitrofen rat model of CDH suggest that the liver does not simply herniate into the chest but rather the liver grows into the thorax due to lack of compression by the diaphragm (31). Lastly, fetal ultrasound studies of clinical CDH cases reveal that the herniated liver molds to the surrounding chest wall and contains a unique vasculature to support the altered form (32, 33).

The greater liver growth seen in right vs. left sided CDH may have resulted from slower growth of the intrathoracic liver in left-sided CDH due to kinking of the hepatic artery and portal vein. The decreased blood flow to the liver above the diaphragm could result in less liver growth over time (34–36). Fetal liver blood flow affects fetal growth and body composition (37, 38). The fetal liver is oxygenated *via* the hepatic artery, umbilical vein and the portal vein. Prior studies have demonstrated that increasing blood flow from the umbilical vein leads to higher cell proliferation in the liver, heart, skeletal muscle and kidneys in fetal lamb (39). In addition, studies of human low-risk pregnancies have shown that larger liver size is associated with higher umbilical vein flow to the fetal liver (40). Additionally, the ductus venosus seems to be mechanically compressed when the liver is in the chest which is more severe in right sided CDH and may result in increased blood flow to the liver in these cases (41, 42). Differences in compartmental pressures as well as blood flow between right vs. left CDH may impact both intrathoracic and total liver growth.

In this study, CDH fetuses without liver herniation exhibited a positive correlation with lung growth. However, with liver herniation, this relationship was lost and lung growth was poor throughout gestation in liver up hernias. The severity of lung hypoplasia is the critical parameter affecting survival in cases of prenatally diagnosed CDH (43–45). Several static variables for liver growth and lung growth have been correlated. The current findings extend prior observations and illustrate the relationship between the dynamic changes in the liver and lung throughout development in CDH patients (5, 46). When the liver is not growing in the chest, liver and lung growth parallel fetal growth. When the liver is herniated and growing in the chest, this positive correlation is lost and lung growth decreases as liver growth increases.

The interpretation of the current findings must account for a number of limitations. First, this is a retrospective review of clinical MRI exams taken from a research database. As such, only correlative assessments are possible. Second, the rarity of right-sided CDH cases limits the number of patients in the current analysis and thus limits conclusions resulting from comparisons including this group. Third, MRI-derived volumetric measurements may overestimate tissue volume during fetal development when compared to direct measures such as experimental tissue displacement. However, relative volumes for perfused tissue *in situ* may not be adequately captured by analysis after animal necropsy and may be better estimated with live cross-sectional imaging (47). Fourth, liver growth was correlated with lung growth alone. There are arguably several other factors involved including the stomach position. Additionally, other outcomes could be evaluated including growth of the pulmonary vasculature, the trachea and bronchi, the left ventricle and the aortic arch. These disease manifestations are critical outcomes and will be important areas for future study. Finally, inter-reviewer reproducibility was not verified in this study, but has been demonstrated to be moderate-to-good for liver volumes above the diaphragm and good-to-excellent for liver volumes below the diaphragm after standardization (23).

More research is needed to better understand the complex interplay between liver and lung growth in CDH patients. Previous studies in rat embryos have shown that the volume of liver herniated into the chest is associated with reduced lung volume (48). In humans, presence of the liver above the diaphragm is a marker of increased severity; however, herniation of the liver in right sided CDH is almost always present and may carry less prognostic significance (19, 22, 49).

The current findings illustrate the potential for liver growth to influence lung growth in CDH fetuses. Future studies incorporating liver and stomach growth in relation to MRI-measured growth of: (1) the pulmonary vasculature; (2) the trachea and bronchi; (3) the left ventricle; and (3) the aortic arch will better characterize the broader impact of liver growth and stomach position on the severity of pulmonary hypoplasia, pulmonary hypertension, tracheobronchomalacia, small left-sided heart structures and aortic arch hypoplasia. Ultimately, these disease manifestations are the critical outcomes that may be affected by growth of the abdominal viscera in the fetal chest.

## Conclusion

The current study utilized serial MRI volumetrics to demonstrate that the side of the hernia results in different liver growth trajectories above the diaphragm as well as overall changes in total liver growth. Additionally, when the liver is not growing in the chest, the liver and lung growth correlate throughout gestation as the fetus grows. But, when the liver is herniated and growing in the chest, this correlation is lost and lung growth actually decreases as liver growth increases. The greater significance of the interplay between liver and lung growth will guide future studies to explain how the side of the diaphragmatic hernia as well as altered compartmental pressure and liver blood flow regulate the molecular and cellular mechanisms of liver and lung growth in CDH fetuses.

## Data availability statement

The raw data supporting the conclusions of this article will be made available by the authors, without undue reservation.

## Ethics statement

The studies involving human participants were reviewed and approved by the institutional review board at Cincinnati Children's Hospital Medical Center as well as Ann and Robert H. Lurie Children's Hospital. The patients/participants provided their written informed consent to participate in this study.

## Author contributions

All persons who meet authorship criteria are listed as authors, and certify that they have participated sufficiently in the work to take public responsibility for the content, including participation in the concept, design, analysis, writing, or revision of the manuscript. All authors contributed to the article and approved the submitted version.

## Funding

The authors declare that the research was conducted in the absence of any commercial or financial relationships that could be construed as a potential conflict of interest.

## Conflict of interest

The authors declare that the research was conducted in the absence of any commercial or financial relationships that could be construed as a potential conflict of interest.

## Publisher's note

All claims expressed in this article are solely those of the authors and do not necessarily represent those of their affiliated organizations, or those of the publisher, the editors and the reviewers. Any product that may be evaluated in this article, or claim that may be made by its manufacturer, is not guaranteed or endorsed by the publisher.
